# Comparative transcriptome analysis uncovers cell wall reorganization and repressed cell division during cotton fiber initiation

**DOI:** 10.1186/s12861-021-00247-3

**Published:** 2021-10-29

**Authors:** Wenyuan Liu, Yanjia Lv, Xiaoyue Li, Zongqin Feng, Lichen Wang

**Affiliations:** College of Life Science, Linyi University, Linyi, 276000 Shandong China

**Keywords:** Lint fiber initiation, Transcriptome sequencing, *Xuzhou142 fibreless* mutant (*Xu142fl*), *MML*, *EXPA*

## Abstract

**Background:**

Tetraploid cotton plants serve as prime natural fiber source for the textile industry. Although various omics studies have revealed molecular basis for fiber development, a better understanding of transcriptional regulation mechanism regulating lint fiber initiation is necessary to meet global natural fiber demand.

**Results:**

Here, we aimed to perform transcriptome sequencing to identify DEGs (differentially expressed genes) in ovules of the cotton variety Xu142 and its fibreless mutant *Xu142fl* during early lint fiber initiation period. Totally, 5516 DEGs including 1840 upregulated and 3676 downregulated were identified. GO enrichment analysis revealed that the downregulated DEGs were mainly associated with biological processes such as transcription related biosynthesis and metabolism, organic cyclic compound biosynthesis and metabolism, photosynthesis, and plant cell wall organization, with molecular functions involving transcription related binding, organic cyclic compound binding, and dioxygenase activity, while the upregulated DEGs were associated with DNA replication and phospholipid biosynthetic related processes. Among the 490 DEGs annotated as transcription factor genes, 86.5% were downregulated in the mutant including the *Malvaceae*-specific *MMLs*, expression patterns of which were confirmed during the central period of lint fiber initiation. Investigation of the 16 genes enriched in the cell wall organization revealed that 15 were EXPA coding genes.

**Conclusions:**

Overall, our data indicate that lint fiber initiation is a complicated process involving cooperation of multiple transcription factor families, which might ultimately lead to the reorganization of the cell wall and terminated cell division of the differentiating fiber initials.

**Supplementary Information:**

The online version contains supplementary material available at 10.1186/s12861-021-00247-3.

## Background

Cotton plants serve as the largest natural fiber source for the global textile industry [1]. Mature cotton seeds are covered with adherent fuzz and spinnable lint fibers. Cotton fibers development can be classified into four overlapping stages: initiation, elongation, thickening of the secondary cell wall, and maturation [2]. Lint fibers start initiation from − 3 DPA and continue to 3 DPA [3]. The initiation of lint fiber involves multiple regulators such as the upstream transcription factors and the downstream expansins proteins [4–6].

The transcriptional mechanisms have been widely explored by various researchers, which established the model in cotton that fiber initiation mimics the hair trichome initiation in *Arabidopsis* involving the MBW complex consisting of the R2R3 MYB protein GL1, the bHLH protein GL3, and the WD-repeat containing protein TTG1, which controls the expression of the downstream HD-ZIP transcription factor gene *GL2* [7]. In cotton, the *GL1* homologous R2R3-MYB transcription factor genes *GhMYB25-like* and *GhMYB25* have been implicated in regulating fiber initiation and elongation respectively [8, 9], and renamed as *MYB-MIXTA-like 3* (*MML3*) and *MML7* respectively lately [10]. Previously, the *N1* gene (*GhMML3_A12*) in *N1 naked seed mutant* (*N1NSM*) and the *li3* gene (*GhMML4_D12*) in *Xuzhou142 fibreless mutant* (*Xu142fl*) have been isolated through a map based cloning method respectively [11, 12]. Totally 10 MMLs in *Gossypium raimondii* have been classified into one lineage as the *Malvaceae*-specific 9th subfamily R2R3-MYBs that regulates epidermal cell differentiation [12], different from the 15th subfamily which regulates leaf hair trichome development in *Arabidopsis* according to evolution analysis [13], and they contain a signature protein motif and are highly expressed during the lint fiber initiation period [10]. Other homologous transcription factor genes in the MBW pathway such as *GL3* homolog bHLH transcription factor gene *GhDEL65* [14] and *GL2* homologs *GaHOX1* and *GhHOX3* also contribute to lint fiber development [15, 16].

The down-stream biological events regulating cell wall reorganization and biosynthesis employ various proteins like expansins, sucrose synthases, and tubulins [17–19]. Expansins are the first identified cell-wall-loosening proteins [20], which function by weakening the noncovalent bonds between cell wall matrix polymers to promote slippage of cellulose microfibrils and cause cell wall relaxation and cell extension [21], and constitute a large multigene family of four groups: alpha-expansin (EXPA), beta-expansin (EXPB), expansin-like A, and expansin-like B. EXPAs were firstly speculated to involve in fiber development since the isolation of two alpha-expansin cDNAs from the developing fiber of *Gossypium hirsutum* [22], then two homologous fiber-specific EXPAs coding genes-*GbEXPA2* from the D subgenome and *GbEXPATR* from the A subgenome of *Gossypium barbadense* were cloned and genetically characterized to participate in the process of lint fiber elongation, evidenced by the facts that silencing of *GbEXPA* in *G. hirsutum* resulted into shorter fibers with thicker cell walls while overexpressing *GbEXPATR* induced longer, finer, and stronger fibers coupled with significantly thinner cell walls [23]. It has been reported that overexpression of *GhEXPA8* significantly improves fiber length and micronaire value [24], while co-expression of *GhRDL1* and *GhEXPA1* led to longer fiber [25]. However, the correlation between expansins proteins and lint fiber initiation at the transcriptome level still need to be investigated.

Despite various studies strongly suggested the key role of Xu142 and *Xu142fl* in investigating lint fiber initiation; however, transcriptional regulation mechanisms of Xu142 and *Xu142fl* regulating fiber initiation, in particular, with a focus on the changing upstream transcription regulations and the downstream cell wall reorganization during early lint fiber initiation in cotton are poorly understood. In this study, we compared the whole genome transcription changes between Xu142 and *Xu142fl* at early fiber initiation stages and identified 3676 downregulated and 1840 upregulated genes. Gene Ontology (GO) enrichment analysis revealed that the downregulated genes were mainly involved in the biological processes related to transcription, organic cyclic compound biosynthesis and metabolism, photosynthesis, response to chitin and plant cell wall organization, and had molecular functions such as transcription related binding, organic cyclic compound binding and dioxygenase activity, while the upregulated DEGs were enriched in DNA replication and phospholipid biosynthesis related processes. Classification of the transcription factor genes revealed that various transcription factor families involved in lint fiber initiation, including the members belonging to the MBW complex. Examination of the cell wall organization related genes revealed 15 EXPA coding genes and one pectinesterase (PE) coding gene, which may contribute to cell loosening and reorganization during lint fiber initiation. Together, these findings provide preliminary data which suggest importance of introducing the essential transcription factor genes or *EXPAs* for improving lint fiber initiation rate.

## Methods

### Plant materials

One cotton variety *Gossypium hirsutum* cv. Xu142 and two fibreless cotton mutants *Xu142fl* and *n2NSM* were selected in this study. The seeds of these lines were retrieved from National Medium-term Gene Bank of Cotton in China and National cotton germplasm resources platform. The seeds were grown in the experimental field of Linyi University in April 14, 2018. Floral buds at − 3, − 1, 0 and 1 DPA on the 3rd to 5th fruiting branches were collected between July and August during the flowering peak, with ovules dissected from the ovaries in the lab, frozen immediately in liquid nitrogen and stored at − 80 °C.

### Transcriptome sequencing and bioinformatics analysis

High-quality RNA extraction was performed from the frozen ovules tissues collected at − 3 DPA and − 1 DPA of Xu142 and *Xu142fl* respectively as previously reported [26]. Subsequent cDNA libraries were constructed and sequenced with BGI-SEQ500 at the Beijing Genomics Institute (BGI, Shenzhen, China).

The raw reads were filtered first to get clean data, and then aligned to the genome of *G. hirsutum* L. (https://cottonfgd.org/about/download/assembly/genome.Ghir.NAU.fa.gz) to reconstruct transcripts through String Tie (http://ccb.jhu.edu/software/stringtie, v1.0.4), identify known genes by Bowtie2 (http://bowtie-bio.sourceforge.net/Bowtie2, v2.2.5) [27], and predict new transcripts using HISAT2 (http://www.ccb.jhu.edu/software/hisat, v2.0.4) [28].

Expression levels of genes and transcripts were calculated using RSEM [29], and expression corrections were calculated by Cor package of R (v3.6.2). DEGs (filtered by fold change ≥ 2, Q-value ≤ 0.001) between Xu142 and *Xu142fl* were identified through DEGseq [30], after normalizing raw reads of each gene as Fragments Per Kilobase of transcript per Million mapped reads (FPKM). GO enriched terms were determined by Q-value ≤ 0.001 using the phyper package of R.

The transcription factor (TF) coding genes were predicted by using Getorf (http://emboss.sourceforge.net/apps/cvs/emboss/apps/getorf.html) to get the ORF of all unigenes first, and then aligned to TF protein structure domain using hmmsearch (http://hmmer.org, v3.0) to annotate TF through the property of specific TF family described in PlantTFDB (http://planttfdb.cbi.pku.edu.cn, v5.0). Heatmaps of TF proteins were generated by Genesis v1.7.6 [31].

### RT-PCR and RT-qPCR analyses

RT-PCR and qRT-PCR were used to evaluate expression levels of *GhMMLs*. Total RNA from ovules at − 1, 0 and 1 DPA of *n2NSM* and *Xu142fl* was extracted as previously reported [26]. Subsequent cDNA was synthesized using RevertAid First Strand cDNA Synthesis Kit (Thermo Scientific, USA) according to the manufacture’s instruction. Total 20-µl-reaction volume was applied for RT-PCR analysis to evaluate expression levels of *GhMMLs*. After this, PCR reaction mixture was subjected to 95 °C denaturation for 3 min, then 29 cycles of amplification of the endogenous reference gene *GhUbq7* or 38 cycles for *GhMMLs* at 95 °C for 30 s, 55 °C annealing for 30 s and 72 °C extension for 30 s, plus a final extension at 72 °C for 5 min. Quantitative real-time PCR (qRT-PCR) was carried out using Hieff qPCR SYBR Green Master Mix (No Rox) (Yeason, Shanghai, China). The calculation of the relative expression levels of each gene and statistical analysis were based on at least three biological and three technical replicates, and the significance was determined by multiple comparisons using Statistix software (version 8.0). Primers were designed by Primer 5.0 and synthesized commercially (Genscript Bioscience, Nanjing, China). The sequences of all the primers sequences are listed in Additional file 1: Table S1.

## Results

### Overview of the comparative transcriptome sequencing using ovules of *Xu142fl* and Xu142 at early fiber initiation stage

In order to study *Xu142fl* and Xu142 in the context of fiber initiation, we first performed transcriptome sequencing by mixing ovules at − 3 and − 1 DPA of *Xu142fl* and Xu142 respectively, before obvious fiber initials could be observed from the epidermis of the wild type cotton seeds under optical microscope, by setting 3 biological replicates for each variety. After removing adapter contamination and low quality tags, a total of 66.12–72.19 million clean reads were generated from each library, with clean read ratios between 92.51–93.74%, and ~ 95% of the clean reads can be mapped to cotton TM-1 genome (Table 1). Besides this, 49,381 novel transcripts were identified, including 36,093 candidate protein coding and 13,288 noncoding transcripts and 5604 novel genes were predicted.

**Table 1:** Overview of the data quality and genome mapping of the transcriptome sequencing of Xu142 and *Xu142fl*

Ovule sample	Total raw reads (M)	Total clean reads (M)	Clean reads Q20 (%)	Clean reads ratio (%)	Total mapped (%)	Uniquely mapped (%)
Xu142-1	70.82	66.39	98.56	93.74	95.89	76.20
Xu142-2	75.39	70.42	98.59	93.41	95.55	75.11
Xu142-3	73.31	68.7	98.59	93.71	95.95	75.99
*Xu142fl*-1	70.61	66.12	98.53	93.64	95.75	74.58
*Xu142fl*-2	75.72	70.78	98.55	93.48	95.85	75.43
*Xu142fl*-3	78.04	72.19	98.6	92.51	95.87	75.52

*M* megabase

After calculating the expression levels of each gene in each sample; Pearson correlation (R^2^) was calculated based on the whole gene expression profile between each sample pair among the total 6 samples. The result showed that the correlations between biological repeats were 0.966–0.993 for the mutant pairs, and 0.971–0.996 for the wild type pairs, but lower (0.921–0.960) between mutant and wild type pairs (Additional file 2: Fig. S1). The results indicated high uniformity between biological repeats. Finally, 5516 DEGs including 1840 upregulated and 3676 downregulated genes were identified in the mutant compared with the wild type.

### Complicated upstream transcription and downstream biosynthesis and metabolism events occurred during early lint fiber initiation

**Fig. 1:**
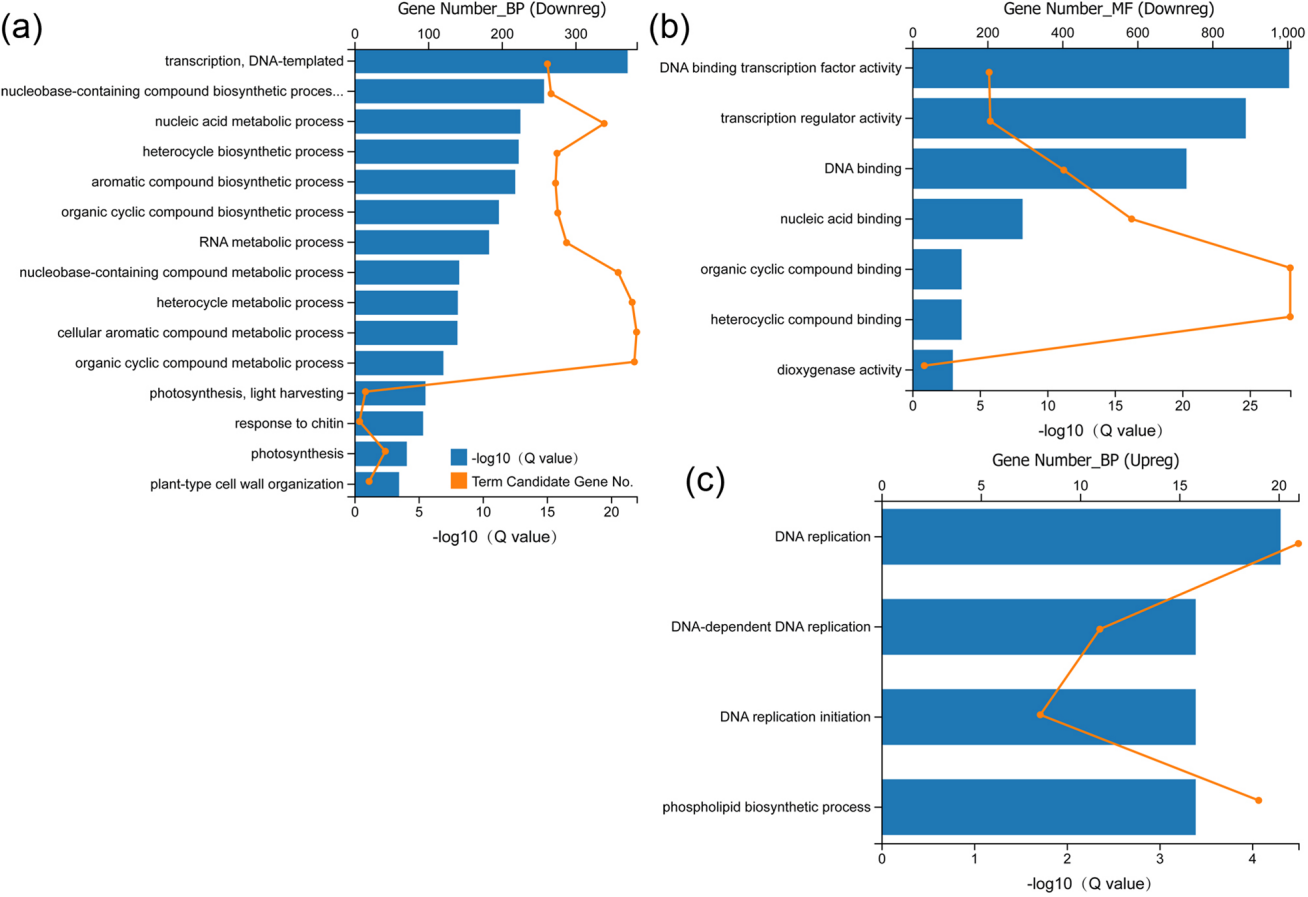
Significantly enriched GO terms on level 3 of the down- and upregulated genes respectively. Graphs showing functional annotation of GO terms and gene number for the downregulated genes on the level of biological process (**a**) and molecular function (**b**), and for the upregulated genes on the level of biological process (**c**). The blue bars showing − log10(Q value) of the enrichment analysis of each term, and orange dots showing the gene number contained by each GO term. *Upreg* upregulated genes, *Downreg* downregulated genes, *BP* biological process, *MF* molecular function

To investigate the biological processes and functions attributed to the DEGs, GO enrichment analyses of the downregulated and upregulated DEGs were conducted respectively. The results showed that the downregulated genes were enriched in 15 level 3 biological process (BP) terms including transcription (262 genes), RNA (288 genes) and nucleic acid metabolism (339 genes), nucleobase-containing compound biosynthesis (267 genes) and metabolism (358 genes), heterocycle biosynthesis (275 genes) and metabolism (377 genes), aromatic compound biosynthesis (273 genes) and metabolism (383 genes) and organic cyclic compound biosynthesis (276 genes) and metabolism (380 genes), photosynthesis (42 genes) and light harvesting (15 genes), respond to chitin (7 genes), and plant cell wall organization (20 genes) (Fig. 1a; Additional file 3: Table S2), with molecular functions (MF) including DNA binding transcription factor activity (205 genes), transcription regulator activity (208 genes) and dioxygenase activity (32 genes), and DNA (404 genes), nucleic acid (585 genes), organic cyclic compound and heterocyclic compound binding activities (1008 genes) (Fig. 1b; Additional file 4: Table S3). These results demonstrated that the essential transcriptional regulations in *Xu142fl* were impaired and led to the aborted lint fiber initiation.

Compared with the significantly enriched GO terms for the downregulated DEGs, the enriched GO terms for the upregulated DEGs were fewer on the BP level, and no GO terms were found on the MF level. The significantly enriched level 3 terms was DNA replication (21 genes), DNA-dependent DNA replication (11 genes), DNA replication initiation (8 genes) and phospholipid biosynthesis (19 genes) (Fig. 1c; Additional file 5: Table S4). After removing redundancy and genes without annotation, 20 DNA replication related genes encoding proteins such as DNA replication licensing factors MCMs and Cell division control proteins CDCs, and 5 phospholipid biosynthesis related genes encoding phosphoethanolamine *N*-methyltransferase 1 (NMT1), inositol-3-phosphate synthase (IPS), choline/ethanolaminephosphotransferase 1 (AAPT1), AAPT2, and phosphatidate cytidylyltransferase (TAMM41) were found (Additional file 6: Table S5). Together, these findings suggest that DNA replication and phospholipid biosynthesis in the process of cell mitosis division were inhibited in the fiber initials of wild type during lint fiber initiation.

### Transcription factor expression dynamics during early fiber initiation

**Fig. 2:**
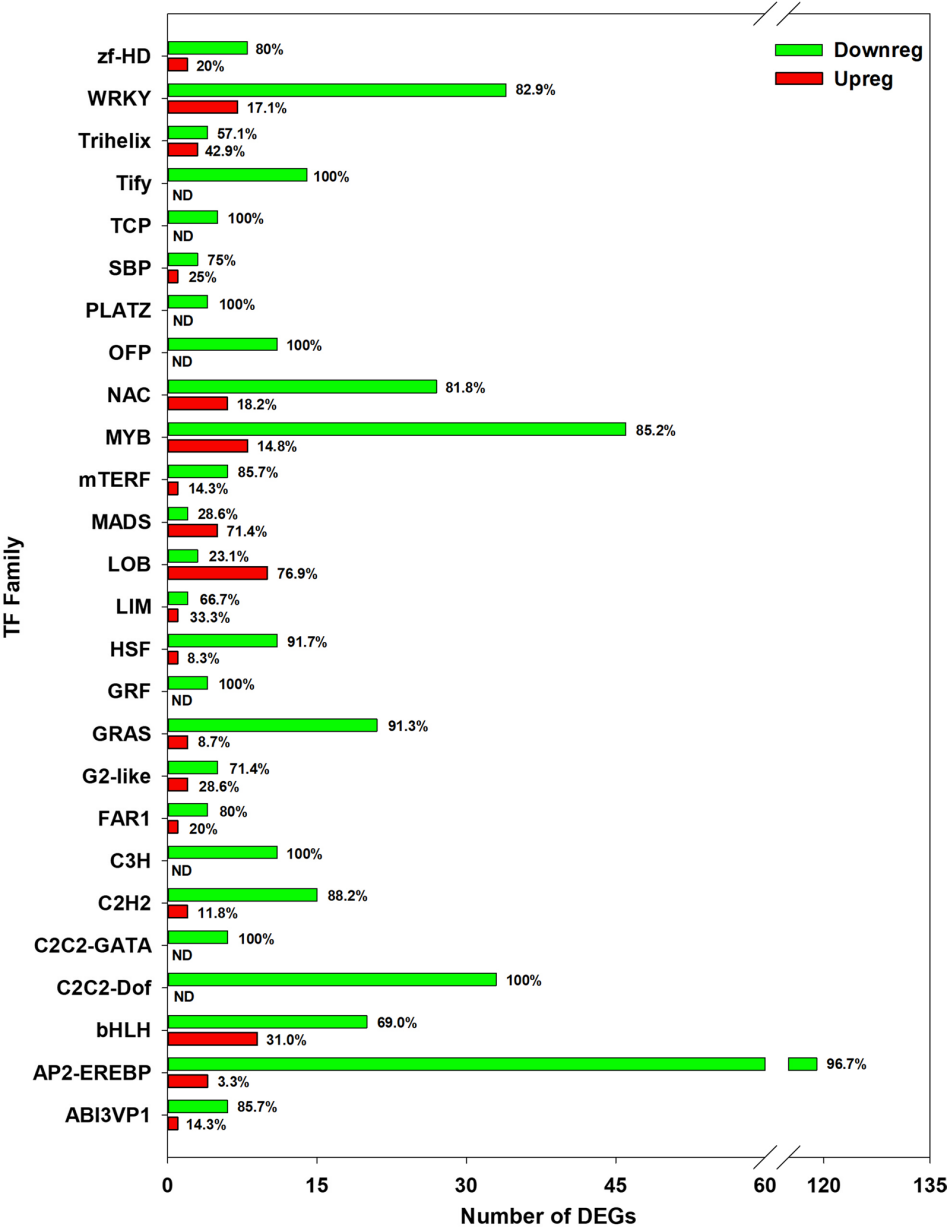
Statistics of the differentially expressed transcription factor genes. The graph showing the number and proportions of the downregulated (green bars, shown as ‘Downreg’) and upregulated DEGs (red bars, shown as ‘Upreg’) belonging to each TF family, with the percentage proportions noted above each bar. ND means ‘not detected’

Next, to characterize the complicated transcription regulations during lint fiber initiation, the TF coding genes were firstly predicted and then filtered to obtain the differentially expressed TF genes closely related to lint fiber initiation (only TF family containing more than two genes were considered), and finally 490 genes belonging to 26 TF families were identified, consisting of 424 downregulated genes and 66 upregulated genes. Moreover, most TF families consisted more downregulated genes (57.1–100%), except for LOB domain-containing and MADS domain-containing families which contained more up-regulated genes (76.9% and 71.4% respectively) (Fig. 2).

**Fig. 3:**
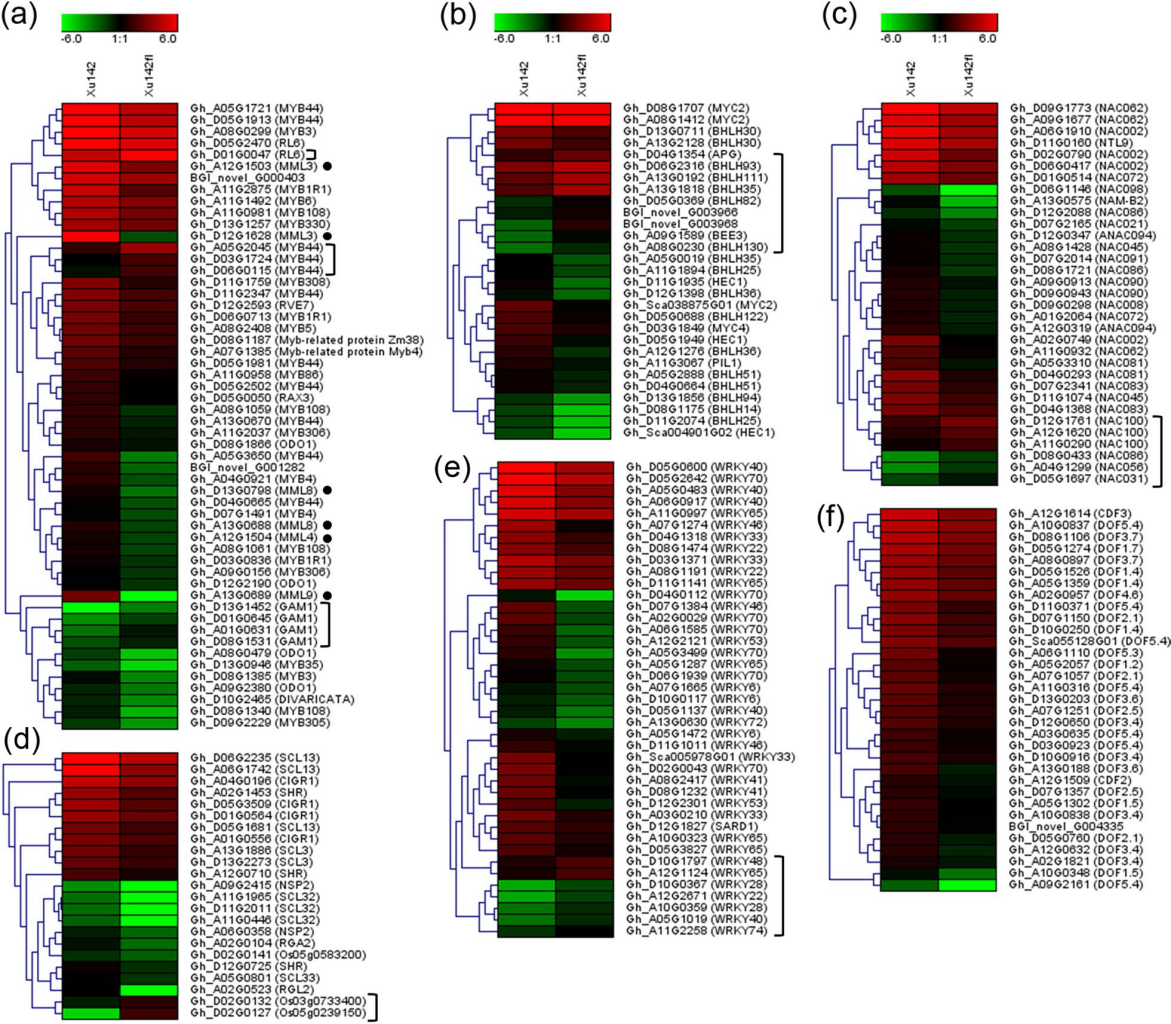
Expression patterns of 6 transcription factor family genes in ovules of Xu142 and *Xu142fl*. Heat maps of MYB (**a**), bHLH (**b**), NAC (**c**), GRAS (**d**), WRKY (**e**), and DOF (**f**) domain containing transcription factor genes. The graphs were generated by Genesis which showing the hierarchical clusters of each type of genes. *MMLs* homologs in (**a**) were indicated with black dots, and the upregulated genes in (**a**)–(**e**) were marked using a bracket. The log2 values of the RPKM of each gene were used to plot the map, which were indicated by the gradient color bars (red to green reflecting the expression levels from high to low)

As shown in Fig. 2, among the down-regulated TF families containing members more than 20, the biggest TF family was AP2-EREBP, which contained 123 DEGs, followed by MYB (54 DEGs), WRKY (41 DEGs), NAC (33 DEGs), C2C2-Dof (33 DEGs), bHLH (29 DEGs) and GRAS (23 DEGs). Subsequently, we selected six TF families including MYB, bHLH, NAC, C2C2 Dof, GRAS, and WRKY containing members ranging from 23–54 to plot expression heat maps. The results showed that 6 *Malvaceae*-specific *MML homologs* [12] including two *MML3*, one *MML4*, two *MML8* and one *MML9* were all downregulated. The *MML3* on chromosome D12 chromosome (Gh_D12G1628) had higher expression level in the wild type and was much more downregulated in the mutant than that one on A12 chromosome (Gh_A12G1503) (Fig. 3a). Although most members were downregulated in the identified TF families, subclasses of members were still upregulated for 5 TF families, including the MYB family which contains one *RL6*, 3 *MYB44* and 4 *GAM1* (Fig. 3a), bHLH family which contains 5 members including *BHLH82*, *BHLH130*, *BEE3* and two novel genes (Fig. 3b), NAC family which contains 6 *NAC* genes, three of which annotated as *NAC100* had dominant expression in both varieties (Fig. 3c), the GRAS family which contains 2 members (Fig. 3d), and WRKY family which contains 7 members, with the genes annotated *WRKY48* and *WRKY65* had higher expression levels while expression of the other five genes were lower in both the varieties (Fig. 3e), except for the C2C2 Dof family genes which were all down-regulated (Fig. 3f).

### Main *GhMMLs* contributed to lint fiber initiation

**Fig. 4:**
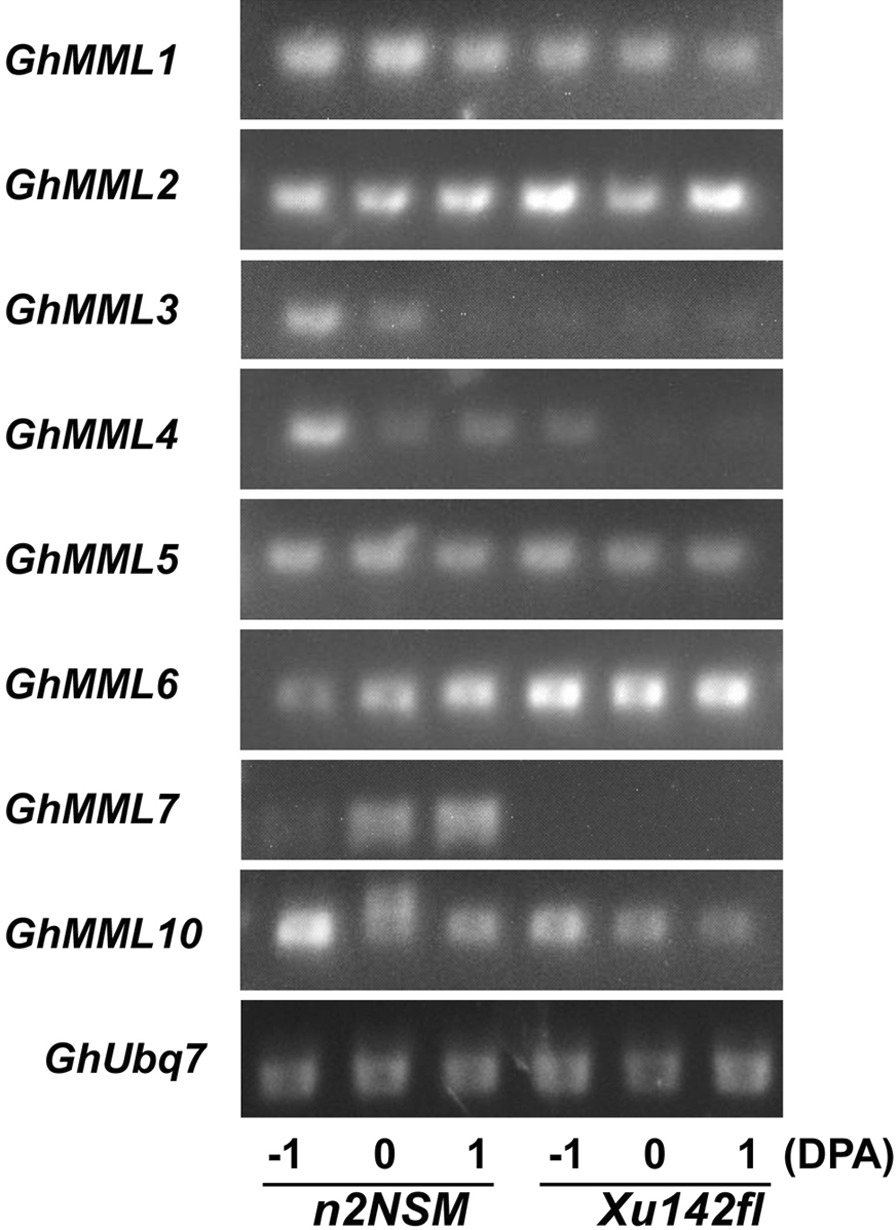
Expression patterns of 8 *GhMMLs* in ovules of *n2NSM* and *Xu142fl* during lint fiber initiation. The transcripts of *GhMML1-7* and *GhMML10* in − 1, 0 and 1 DPA ovules of *n2NSM* and *Xu142fl* were detected by RT-PCR. *GhUbq7* was used as an endogenous reference gene. The original gel pictures are shown in Additional file 7: Fig. S2

**Fig. 5:**
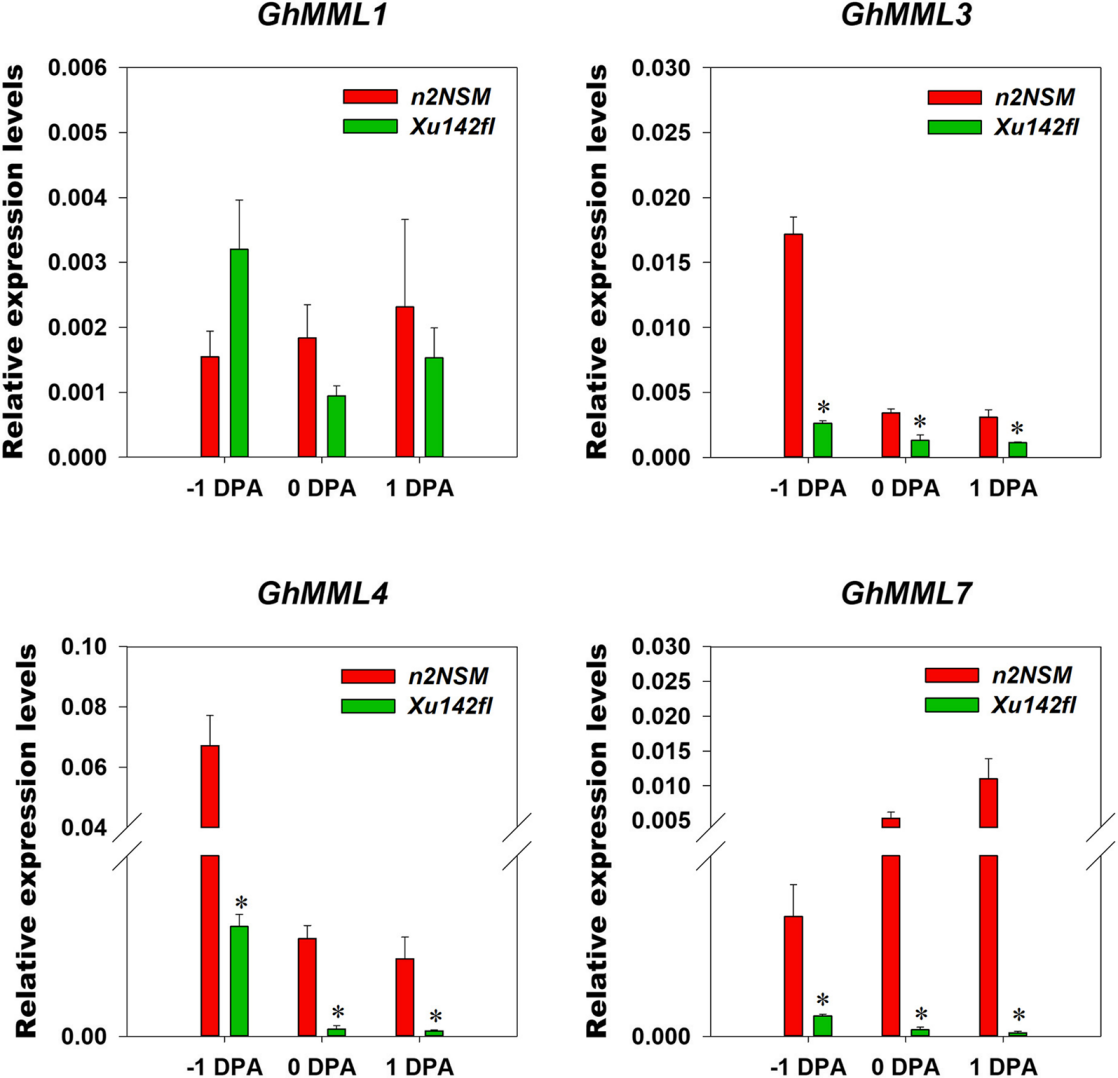
Expression patterns of *GhMML1/3/4/7* in ovules of *n2NSM* and *Xu142fl* during lint fiber initiation. qRT-PCR analysis showing relative expression levels of *GhMML1*, *GhMML3*, *GhMML4* and *GhMML7* in − 1, 0 and 1 DPA ovules of *n2NSM* and *Xu142fl*. *GhUbq7* was used as an endogenous reference gene, and the data represents the mean ± SD of three biological replicates. “*” represent *p* < 0.05

The 9th subfamily R2R3-MYB transcription factors MMLs is considered as *Malvaceae*-specific through evolutionary analysis [12] and among the 10 GhMMLs from GhMML1 to GhMML10, GhMML3 and GhMML4 had been demonstrated responsible for fuzz and lint fiber initiation respectively [11, 12]. Our TF classification revealed that *GhMML3*, *GhMML4*, *GhMML8*, and *GhMML9* were involved in lint fiber initiation, to confirm that, we conducted RT-PCR of all the ten *GhMMLs* in *n2NSM* and *Xu142fl* during early lint fiber initiation period from − 1 DPA to 1 DPA, given the fact that *n2NSM* and *Xu142fl* are all naked seed mutants and the only difference is whether the lint fiber initiates or not [12]. The results showed that 8 *MMLs* can be detected except *GhMML8* and *GhMML9* (Fig. 4, Additional file 7: Fig. S2), and 4 were down-regulated including *GhMML1*, *GhMML3*, *GhMML4* and *GhMML7* in *Xu142fl* compared with *n2NSM*. Further investigation of their expression patterns by qRT-PCR confirmed that *GhMML3*, *GhMML4* and *GhMML7* were significantly down-regulated in *Xu142fl* compared with *n2NSM* at all three time points while no obvious expression differences were observed for *GhMML1* (Fig. 5). We also found that expression levels of *GhMML3* and *GhMML4* in *n2NSM* were decreased, while expression level of *GhMML7* was increased from − 1 DPA to 1 DPA, implying different mechanisms between GhMML7, and GhMML3 and GhMML4 (Fig. 5).

### Expansins enriched in cell wall organization may contribute to early lint fiber initiation

**Fig. 6:**
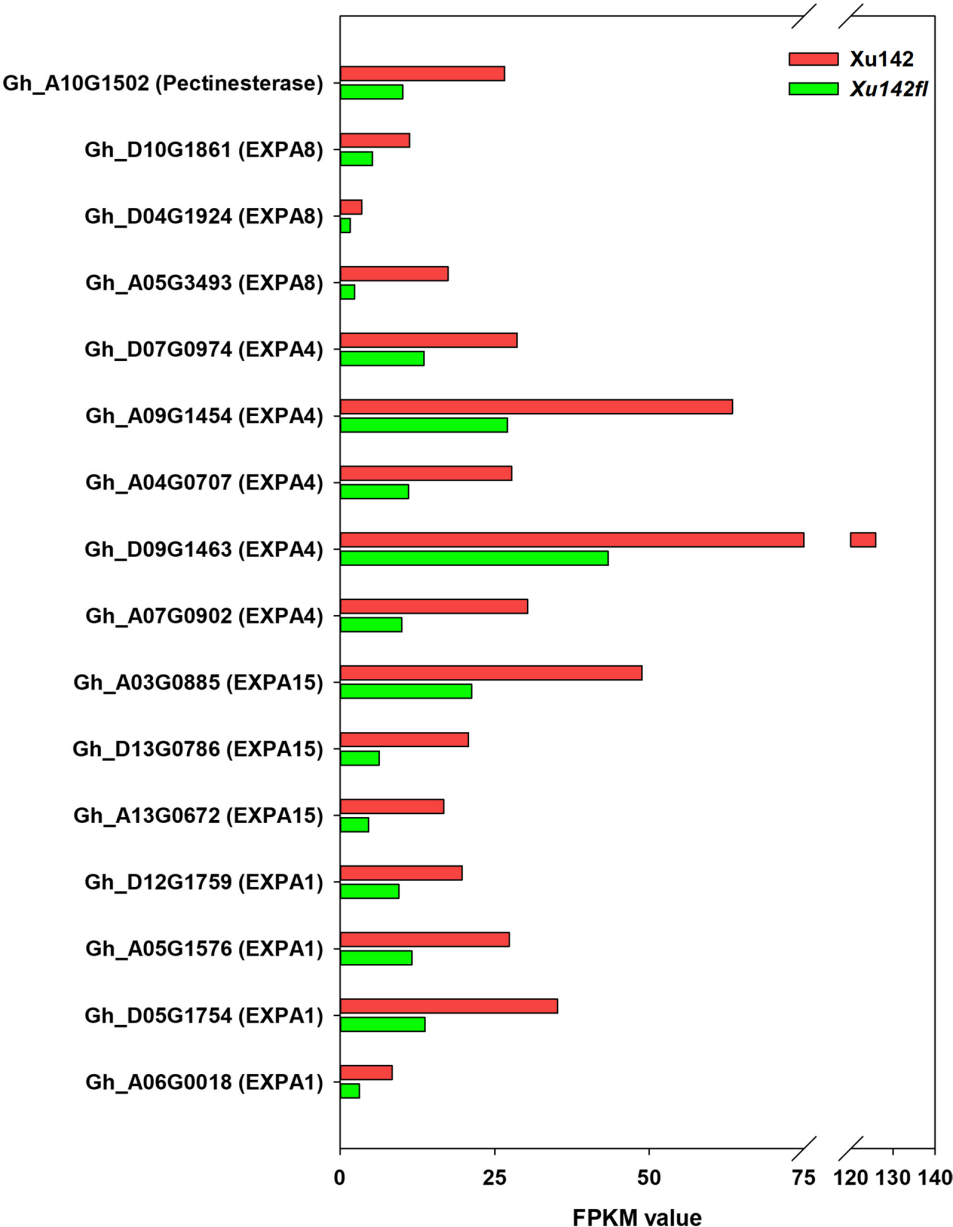
Expression differences of the cell wall reorganization related genes. The graph showing the FPKM values for 15 *EXPAs* and one *PE* in Xu142 (red bars) and *Xu142fl* (green bars)

Cell wall reorganization is an essential event during fiber development involving multiple enzymes and wall proteins [5]. Here, we had a detailed investigation of the GO term-plant cell wall organization which contains 16 genes after removing 4 genes with FPKM < 1 in both lines, and the results showed that 15 were cell wall loosing related alpha-expansin coding genes, including 4 *EXPA1s*, 5 *EXPA4s*, 3 *EXPA8s* and 3 *EXPA15s*, and one was pectinesterase (PE) encoding gene, and they were all downregulated in *Xu142fl* comparing with that in the wild type (Fig. 6). Of special note, data indicated that EXPAs might be the most important cell wall proteins for the early fiber cell initiation.

## Discussion

### Lint fiber initiation is a complicated morphogenesis process involving complex metabolite biosynthesis and metablism

In this study, through GO analysis of the downregulated and upregulated DEGs, multiple enriched biological processes were identified which were associated with lint fiber initiation (Fig. 1). Based on our findings, we speculated that the downregulated DEGs mainly function as positive regulatory factors for lint fiber initiation, while the upregulated DEGs mainly function to inhibit lint fiber initiation.

For the downregulated DEGs, more than 200 genes were associated with DNA-templated transcription (Fig. 1a), which have transcription associated molecular functions as DNA binding transcription factor activity (Fig. 1b). These biological processes related genes may construct the upstream regulation networks during lint fiber initiation, and the downstream regulation involves complicated metabolic cascades associated with organic compound biosynthesis and metabolism including nucleobase-containing compound, organic cyclic compound, heterocyclic compound and aromatic compound such as flavonoid, sugar and phytohormones biosynthesis and metabolism (Fig. 1a, b). Several *NCEDs* (data not shown) which encode the rate-limiting dioxygenases controlling ABA biosynthesis [32], were found in the GO term of dioxygenase (Fig. 1b), implying that ABA might be a positive regulator during lint fiber initiation. This can be supported by the gradually accumulation of endogenous ABA content during the fiber cell initiation and elongation stages [33] although ABA was considered as a negative regulator of fiber initiation [34]. Chitin oligosaccharides can induce various defense responses in a wide range of plant cells including both monocots and dicots [35]. However, defense responses related to fiber initiation still remain unclear. Immature fiber (im) mutant with thinner fiber cell wall compared to the isogenic wild type (TM-1) with fiber of normal thickness revealed that the mutant exhibited lower net photosynthesis, because of the lower chlorophyll content per unit leaf area due to less chlorophyll a levels than that of wild type [36], however how the photosynthesis related genes were changed in *Xu142fl* need further investigation (Fig. 1a).

The upregulated DEGs were mainly enriched in DNA replication and phospholipid biosynthetic processes, which were closely related to cell division. This is reasonable because fiber cells are unable to undergo cell division during fiber development. Before fiber cell differentiation, the ovular epidermal cells are closely packed, cuboidal, and rich in cytoplasm containing a large nucleus, which represents a status of rapid cell division [37]. The early development of fibers consists of two intergrading steps-spherical expansion above the ovular epidermis and elongation [38]. The morphological differentiation of a fiber occurs when an epidermal cell balloons above the epidermal surface, followed by transition to elongation phase and stopping division [39]. Collectively, cell division should be stopped to initiate fiber development in the wild type while rapid cell division continues which inhibit cell differentiation into fiber cells in *Xu142fl* mutant.

### Complicated transcription regulation during lint fiber initiation

In this study, through TF annotation and expression pattern profiling, many down-regulated TF families were identified, including known fiber development related MYBs, bHLHs, and TCPs [6]. Other TF families such as NAC, WRKY, GRAS, and Dof identified here were also detected in another study exploring fiber elongation related pathways by transcriptome analysis of a short fiber mutant and a wild type [40], suggesting that some TF family genes might have dual role both in fiber initiation and elongation. For example, fiber cell expansion and elongation can be mediated by a homeodomain leucine zipper gene, *GhHD-1*, through a WRKY transcription factor by regulating the levels of ethylene and reactive oxidation species (ROS) [41]. Because some lint fiber initiation related TFs which were genetically characterized as positive regulators for fiber development were firstly found downregulated in fiber related mutants, including R2R3 MYB proteins [8, 9, 42, 43], HD-ZIP proteins [15, 16] and bHLH proteins [14], so the other downregulated TF family genes found in this study deserve further investigation of their roles in regulating lint fiber initiation.

Besides this, two class of TF families involved more upregulated DEGs than the downregulated DEGs (Fig. 2), including LOB (Lateral Organ Boundaries) family which are essential in the regulation of plant lateral organ development [44] and MADS which regulate floral organ differentiation and development [45], and small group of genes in other TF families were also upregulated (Figs. 2 and 3), which might be negative regulators of fiber initiation and contribute to the lintless ovules of *Xu142fl* due to their upregulation.

### Dynamic expression of *Malvaceae*-specific *MMLs* during early lint fiber initiation

Among the 10 pair of *Malvaceae*-specific *MYB MIXTA-like* (*GhMML*) homoeologs at least one copy on one sub-genome in allotetraploid cotton were predominantly expressed during fiber initiation in the wild type TM-1 [10], and *GhMML3-A12* and *GhMML4-D12* were demonstrated contributing to the fuzz and lint fiber initiation respectively [11, 12], implying the subfunctionalization of *MML* genes. Combined our transcriptome analysis and RT-PCR analysis, *MML3*, *MML4* and *MML7 should be the MMLs most related to lint fiber initiation* (Figs. 3a, 4 and 5)*.* However, it was contrary in that *GhMML3-D12* (Gh_D12G1628) and *GhMML4-A12* (Gh_A12G1504) might be more important for lint fiber initiation, because *GhMML3-D12* demonstrated higher expression level in the wild type and was more downregulated in the mutant than *GhMML3-A12,* while *GhMML4-D12* was not detected as DEG in our study (Fig. 3a).

Different from *GhMML3* and *GhMML4* which were downregulated in the fuzzless-linted mutant *n2NSM* from − 1 DPA to 1 DPA, *GhMML7* was upregulated and showed the highest expression level in ovules at 1 DPA (Fig. 5). This phenomenon indicated that, except for the role in fiber initiation, *GhMML7* may also involve in fiber elongation, which can be assisted by the evidences that *GhMML7/GhMYB25* expressed in the epidermis of ovules, developing fiber initials and fibers, and *GhMML7/GhMYB25*-silenced cotton produced shorter fiber, while overexpression of *GhMYB25* promoted fiber initiation [8].

### Cell wall organization initiates from the very beginning during lint fiber initiation

Previously, stage-specific developmental markers such as EXPAs, xyloglucan endo-transglycosylases (XETs) and PEs have been reported to regulate fiber cell expansion in cotton [4, 5, 46]. Here, we also identified many EXPA encoding genes and a PE encoding gene, indicated that EXPAs are more important during fiber initiation and elongation. Taken together, upstream regulators of EXPAs will further facilitate our understanding underlying mechanisms of lint fiber initiation and elongation.

## Conclusion

In this study, we compared the whole genome transcription changes between Xu142 and *Xu142fl* at early fiber initiation stages and identified 3676 downregulated and 1840 upregulated genes. GO enrichment analysis revealed that the downregulated genes were mainly involved in the biological processes related to transcription, organic cyclic compound biosynthesis and metabolism, photosynthesis, response to chitin and plant cell wall organization, and had molecular functions such as transcription related binding, organic cyclic compound binding and dioxygenase activity, while the upregulated DEGs were enriched in DNA replication and phospholipid biosynthesis related processes. Classification of the transcription factor genes revealed that various transcription factor families involved in lint fiber initiation, including the members belonging to the MBW complex. Examination of the cell wall organization related genes revealed 15 EXPA coding genes and one PE coding gene, which may contribute to cell loosening and reorganization during lint fiber initiation. Taken together, these findings provide preliminary data which suggest importance of introducing the essential transcription factor genes or *EXPAs* for improving lint fiber initiation rate.

**Fig. 7:**
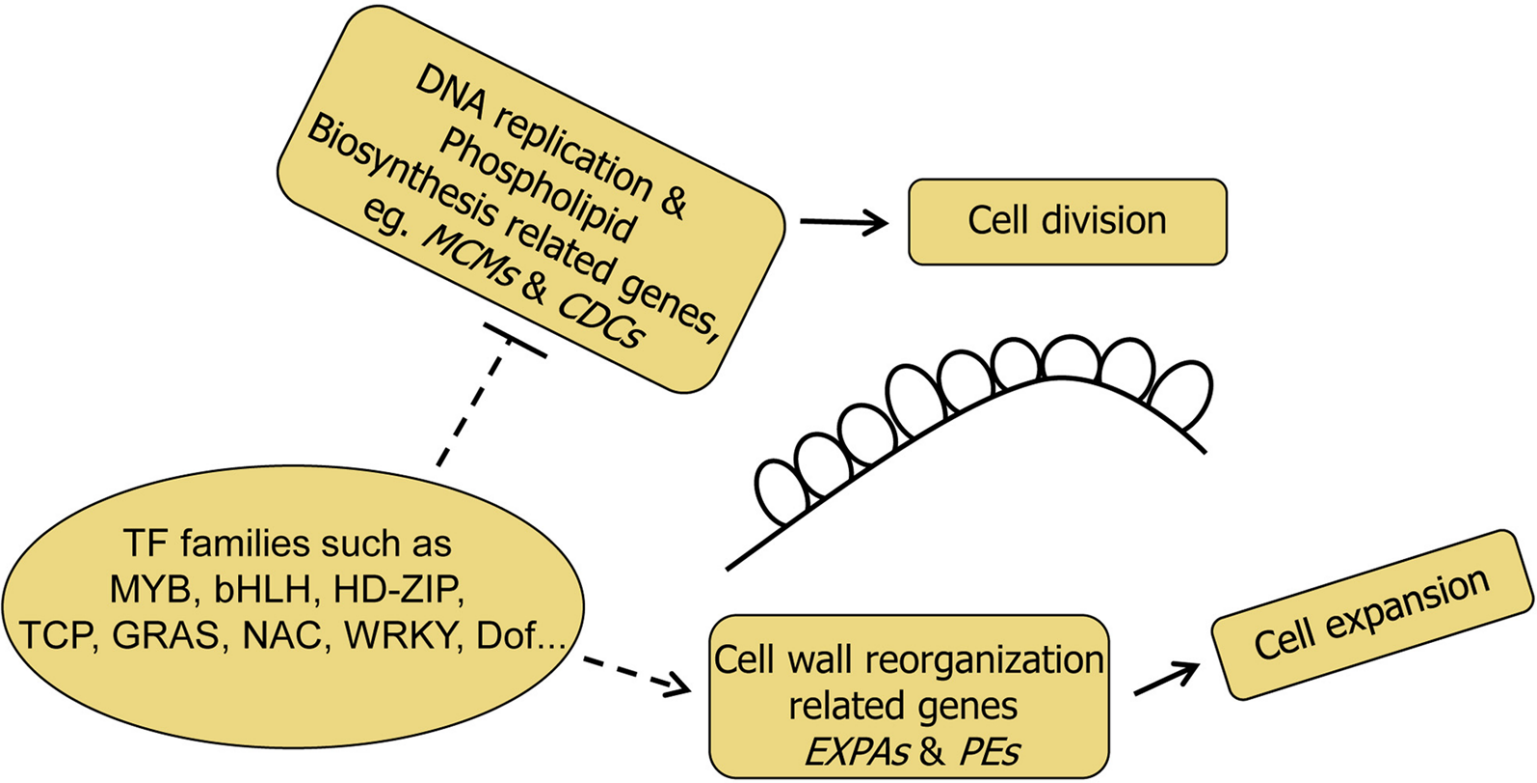
Transcription regulation model of lint fiber initiation in cotton. Multiple TF family proteins are implicated during lint fiber initiation to inhibit expressions of DNA replication and phospholipid biosynthesis related genes such as *MCMs* and *CDCs* to terminate cell division, and activate expression of *EXPAs* and *PEs* to cause cell wall reorganization and fiber cell expansion. *MCMs* DNA replication licensing factors, *CDCs* cell division control proteins, *EXPAs* Alpha-expansin encoding genes; *PEs* Pectinesterase

In conclusion, this study provides novel information for lint fiber initiation mechanism, which may involve dynamic expression of multiple types of TF family genes, which mediate the complicated downstream organic compound biosynthesis and metabolism resulting into the termination of cell division and cell wall reorganization of the expanding fiber cells during lint fiber initiation (Fig. 7).

## Supplementary Information

**Additional file 1: Table S1.** Primers used in this study.

**Additional file 2: Figure S1.** Correlation analysis based on the whole expression profiles of genes from the transcriptome sequencing data in the ovules of Xu142-1-3 and *Xu142fl-1-3*.

**Additional file 3: Table S2.** Enriched level 3 GO terms of the downregulated DEGs in the category of BP.

**Additional file 4: Table S3.** Enriched level 3 GO terms of the downregulated DEGs in the category of MF.

**Additional file 5: Table S4.** Enriched level 3 GO terms of the upregulated DEGs based on the level of BP.

**Additional file 6: Table S5.** Annotations of DNA replication and phospholipid biosynthetic related genes.

**Additional file 7: Figure S2.** RT-PCR analysis of *GhMML1~GhMML10* in ovules of *n2NSM* and *Xu142fl* during lint fiber initiation.

## Data Availability

All the relevant data and Additional files are available including the sequences of the primers used for RT-PCR and RT-qPCR. Raw data for the transcriptomes are available on the GEO platform as series GSE176384.
