# Supplementary material for: Comparative transcriptome analysis uncovers cell wall reorganization and repressed cell division during cotton fiber initiation
Source: BMC Dev Biol. 2021 Oct 29;21:15. doi: 10.1186/s12861-021-00247-3 (PMC8556910; doi:10.1186/s12861-021-00247-3)
Supplement: Supplementary file 2 — Additional file 2: Figure S1. Correlation analysis based on the whole expression profiles of genes from the transcriptome sequencing data in the ovules of Xu142-1-3 and Xu142fl-1-3. [file 12861_2021_247_MOESM2_ESM.pdf]

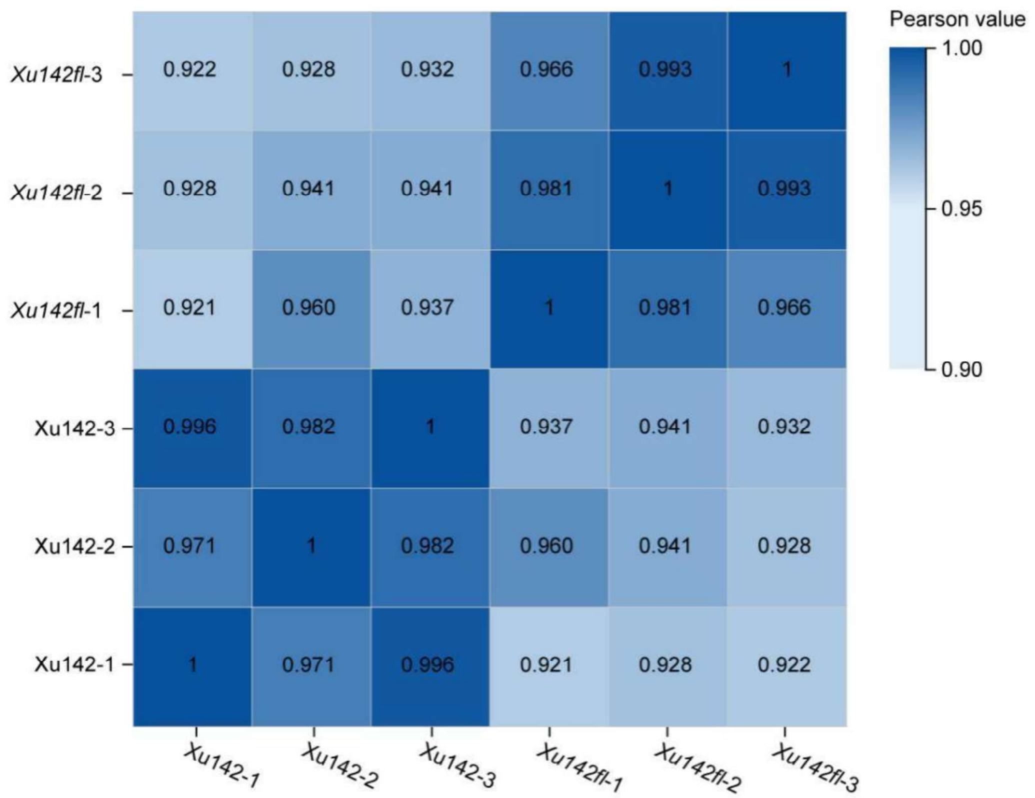

Additional file 2 Figure S1. Correlation analysis based on the transcriptome data of the ovules of Xu142-1~3 and *Xu142fl*-1~3

Pearson values were calculated between each sample pair and were indicated by the gradient blue bar (deep to light reflecting the Pearson values from 1.00 to 0.90).
