# Supplementary material for: Comparative transcriptome analysis uncovers cell wall reorganization and repressed cell division during cotton fiber initiation
Source: BMC Dev Biol. 2021 Oct 29;21:15. doi: 10.1186/s12861-021-00247-3 (PMC8556910; doi:10.1186/s12861-021-00247-3)
Supplement: Supplementary file 7 — Additional file 7: Figure S2. RT-PCR analysis of GhMML1~GhMML10 in ovules of n2NSM and Xu142fl during lint fiber initiation. [file 12861_2021_247_MOESM7_ESM.pdf]

(a)

(b)

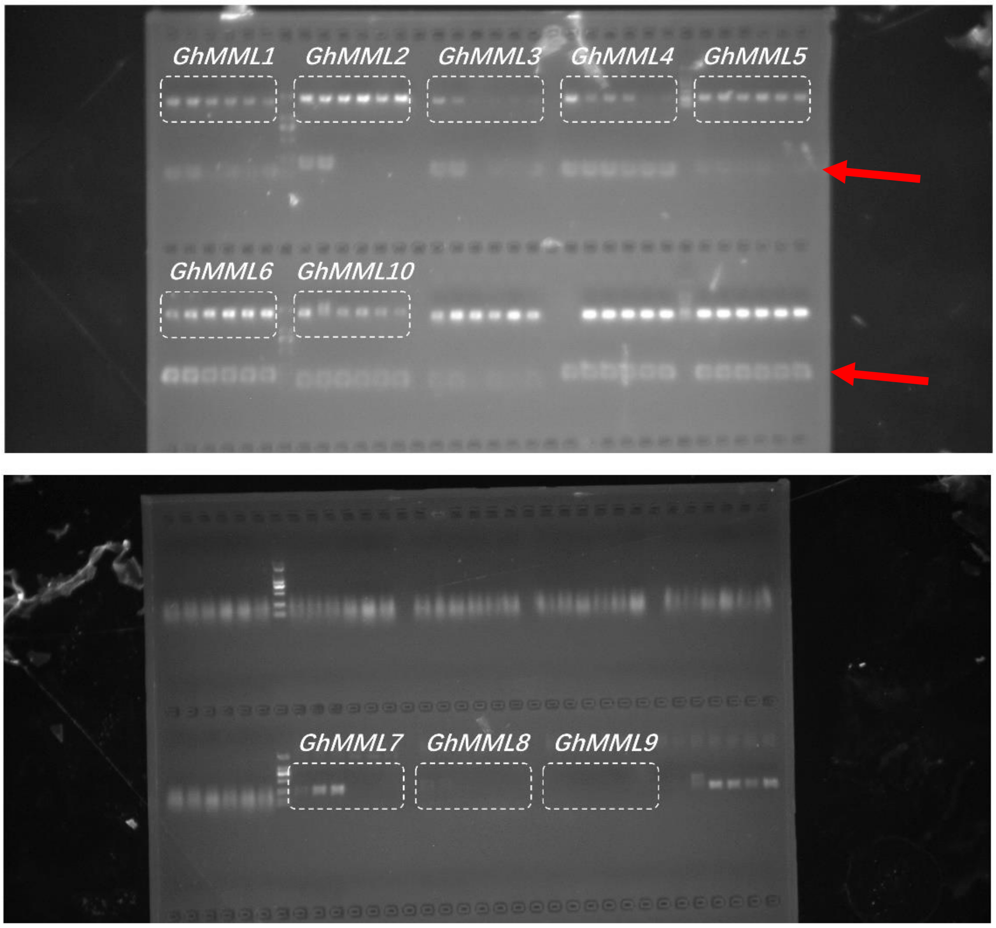

Additional file 7 Figure S2. RT-PCR analysis of *GhMML1~GhMML10* in ovules of *n2NSM* and *Xu142fl* during lint fiber initiation

Agarose gel electrophoresis of RT-PCR products of *GhMML1* ~ *GhMML6*, *GhMML10* (a), and *GhMML7* ~ *GhMML9* (b), in ovule samples of *n2NSM* and *Xu142fl* at -1, 0 and 1 DPA (the arrangement of the blots for each gene is the same as in figure 4). The blots of each of the genes were enclosed in a dotted box, with the name of each gene noted above. The weak signals detected below the *GhMMLs* blots shown by the red arrows are from the last electrophoresis of PCR products for other genes irrelevant to this study.
